# Human mobility and poverty as key drivers of COVID-19 transmission and control

**DOI:** 10.1186/s12889-021-10561-x

**Published:** 2021-03-25

**Authors:** Matan Yechezkel, Amit Weiss, Idan Rejwan, Edan Shahmoon, Shachaf Ben-Gal, Dan Yamin

**Affiliations:** 1 Laboratory for Epidemic Modeling and Analysis, Department of Industrial Engineering, Faculty of Engineering, Tel Aviv University, 6997801 Tel Aviv, Israel; 2 Center for Combatting Pandemics, Tel Aviv University, 6997801 Tel Aviv, Israel

**Keywords:** Contact mixing patterns, Human mobility, COVID-19, Transmission model, SIR model

## Abstract

**Background:**

Applying heavy nationwide restrictions is a powerful method to curtail COVID-19 transmission but poses a significant humanitarian and economic crisis. Thus, it is essential to improve our understanding of COVID-19 transmission, and develop more focused and effective strategies. As human mobility drives transmission, data from cellphone devices can be utilized to achieve these goals.

**Methods:**

We analyzed aggregated and anonymized mobility data from the cell phone devices of> 3 million users between February 1, 2020, to May 16, 2020 — in which several movement restrictions were applied and lifted in Israel. We integrated these mobility patterns into age-, risk- and region-structured transmission model. Calibrated to coronavirus incidence in 250 regions covering Israel, we evaluated the efficacy and effectiveness in decreasing morbidity and mortality of applying localized and temporal lockdowns (stay-at-home order).

**Results:**

Poorer regions exhibited lower and slower compliance with the restrictions. Our transmission model further indicated that individuals from impoverished areas were associated with high transmission rates. Considering a horizon of 1–3 years, we found that to reduce COVID-19 mortality, school closure has an adverse effect, while interventions focusing on the elderly are the most efficient. We also found that applying localized and temporal lockdowns during regional outbreaks reduces the overall mortality and morbidity compared to nationwide lockdowns. These trends were consistent across vast ranges of epidemiological parameters, and potential seasonal forcing.

**Conclusions:**

More resources should be devoted to helping impoverished regions. Utilizing cellphone data despite being anonymized and aggregated can help policymakers worldwide identify hotspots and apply designated strategies against future COVID-19 outbreaks.

**Supplementary Information:**

The online version contains supplementary material available at 10.1186/s12889-021-10561-x.

## Background

Severe acute respiratory syndrome coronavirus 2 (SARS-CoV-2) was identified in Wuhan, China, in December 2019. It has since developed into a pandemic wave affecting over 200 countries, causing over 6.9 million cases and claiming over 390 thousand lives, as of June 8, 2020 [1]. The rapid growth of the SARS-CoV-2 pandemic led to unprecedented control measures on a global scale. Travel bans, restrictions on mobility of varying degrees, and nationwide lockdowns have emerged sharply in over 200 countries [2]. In Israel, since March 9, 2020, travelers from any country are being denied entry unless they can prove their ability to remain under home isolation for 14 days. From March 16 onward, daycare and schools were shut, and work was limited to less than a third of the capacity. On March 26, inessential travel was limited to 100 m away from home, and three separate lockdowns were applied in most regions in Israel to prevent crowding due to holiday celebrations [3].

These massive measures have led to a sharp decline in transmission but pose a significant humanitarian and economic crisis [4–7]. Recent estimates have suggested that 1.5–3 month lockdowns will lead to an enormous economic loss, with high variability across countries ranging between 1.7–13.1% decline in the gross domestic product [4]. Restrictions to mitigate the outbreak also led to various types of psychological distress, including anxiety, helplessness, and depression [5–7]. Furthermore, social isolation is a primary public health concern in the elderly, as it also amplifies the burden of neurocognitive, mental, cardiovascular, and autoimmune problems [7]. Thus, given that pandemics rarely affect all people in a uniform manner [8], it is essential to improve our understanding of the COVID-19 transmission dynamics to customize control efforts.

As human mobility is an intrinsic property of human behavior, it serves as a key component of the transmission of respiratory infections, including COVID-19 [9–13]. The four billion mobile phones in use worldwide are ubiquitous sensors of individuals’ locations and can be utilized not only to track mobility patterns, but also to understand compliance with ongoing restrictions [12]. The importance of human mobility is further intensified by the 2.2–11.5 days of incubation, and the observation that as many as 95% of cases are unreported [14]. Thus, utilizing real-time data on human mobility is instrumental for early detection and prompt isolation of COVID-19 infection.

A variety of factors besides human mobility affect the risk of infection and manifestations, including demographics, education, underlying conditions, and epidemiological characteristics [15]. The high variance in the severity of the disease for different age groups suggests that age-based strategies might be useful in reducing mortality [16]. Age-stratified modeling studies show that interventions such as school closure can help delay the outbreak peak [11]. However, this will not necessarily result in a reduction in the total number of deaths, particularly in light of the estimated time for vaccine availability being > 1 year [17]. In addition to age, individuals with comorbidities are 2.8–21.4 times more likely to become hospitalized following COVID-19 infection [18]. Another factor may be socioeconomic status (SES). Impoverished populations often live in denser regions and have reduced access to health services, thereby being most vulnerable during a crisis [8]. The considerably high rate of household transmission for respiratory infections [19] may also suggest a higher risk for larger families, regardless of lockdowns.

We analyzed a large-scale data of location records from mobile phones to explore the spatiotemporal effect of human mobility and population behavior on transmission. We integrated these mobility data into regional age- and risk-structured transmission model and used our model to identify efficient and effective strategies for reducing COVID-19 mortality. Our methodology can help policymakers worldwide utilize aggregate and anonymized cellphone data to develop designated strategies against future outbreaks.

## Methods

### Human mobility

Our data include mobility records based on cellular data of > 3 million users from one of the largest telecommunication companies in Israel. With the exception of children < 10 years of age, the users are well representative of Israel demographically, ethnically, and socioeconomically. In accordance with the General Data Protection Regulation (GDPR), the data include aggregated and anonymized information. The data specifies movement patterns within and between 2630 zones covering Israel, on an hourly basis, from February 1, 2020, until May 16, 2020. To ensure privacy, if less than 50 individuals were identified in the zone in a given hour, the number of reported individuals was set to zero.

We determined the location of individuals based on the triangulation of cell towers, which was found to be accurate to 300 m in most cases but varied by up to 1 km in less populated areas. To prevent signal noise and identify stay points, we tracked only locations where users stayed for at least 15 min within a distance threshold of 1.5 km. We defined users as residents of a zone based on the location at which they had the highest number of signals on most nights during February 2020. We define a mobility index (MI) as the daily proportion of individuals who traveled > 1.5 km away from their home. To calculate the MI for each zone, we counted the daily number of individuals in each group that showed a signal away from their home location.

**Fig. 1:**
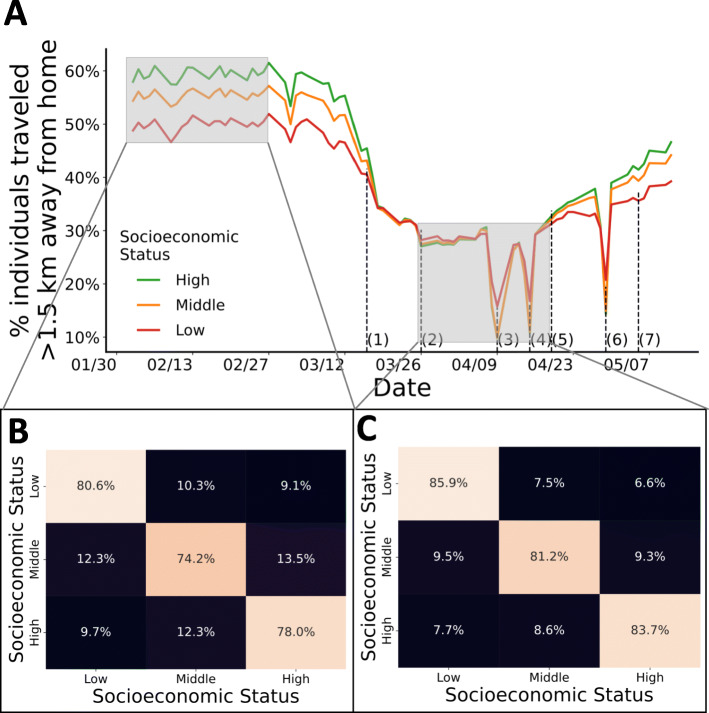
Mobility patterns with and without restrictions. **a** Percentage of individuals who traveled > 1.5 km, stratified by socioeconomic groups, during routine and when mobility restrictions were applied and lifted: (1) closing schools and stores and limiting workplaces to 30% activity; (2) limiting nonessential travels to 100 m away from home; (3, 4) national daily lockdowns due to Passover; (5) opening stores; (6) lockdown due to Independence Day; (7) lifting the 100 m limit for nonessential travels. **b** and (**c**) Travel patterns based on individuals’ SES during February 2–29 (**b**) and March 26–April 18 (**c**)

Next, we integrated data from the Central Bureau of Statistics (CBS) that specifies several socioeconomic characteristics, including population size, household size, age distribution, socioeconomic score, and dominant religion, for each zone. Each zone includes ~ 3500 residents. For each zone, we scaled the number of resident users of the telecommunication company to match the actual number of residents in the zone, as reported by the Israeli CBS. The CBS specifies for each zone a socioeconomic cluster from 1 to 10. Based on these clusters, we defined three SES groups that were nearly equal in size: low (clusters 1–3), middle (clusters 4–7), and high (clusters 8–10). We aggregated the MI according to SES to test the mobility trends on a national level (Fig. 1a). To evaluate the travel patterns based on an individual’s SES (Fig. 1b and c), we counted the mean daily number of travels between the 2630 zones, including for those individuals who stayed in their origin zone. Grouping by SES and scaling the daily number of travels to one for each zone, we created an origin-destination travel probability matrix.

**Fig. 2:**
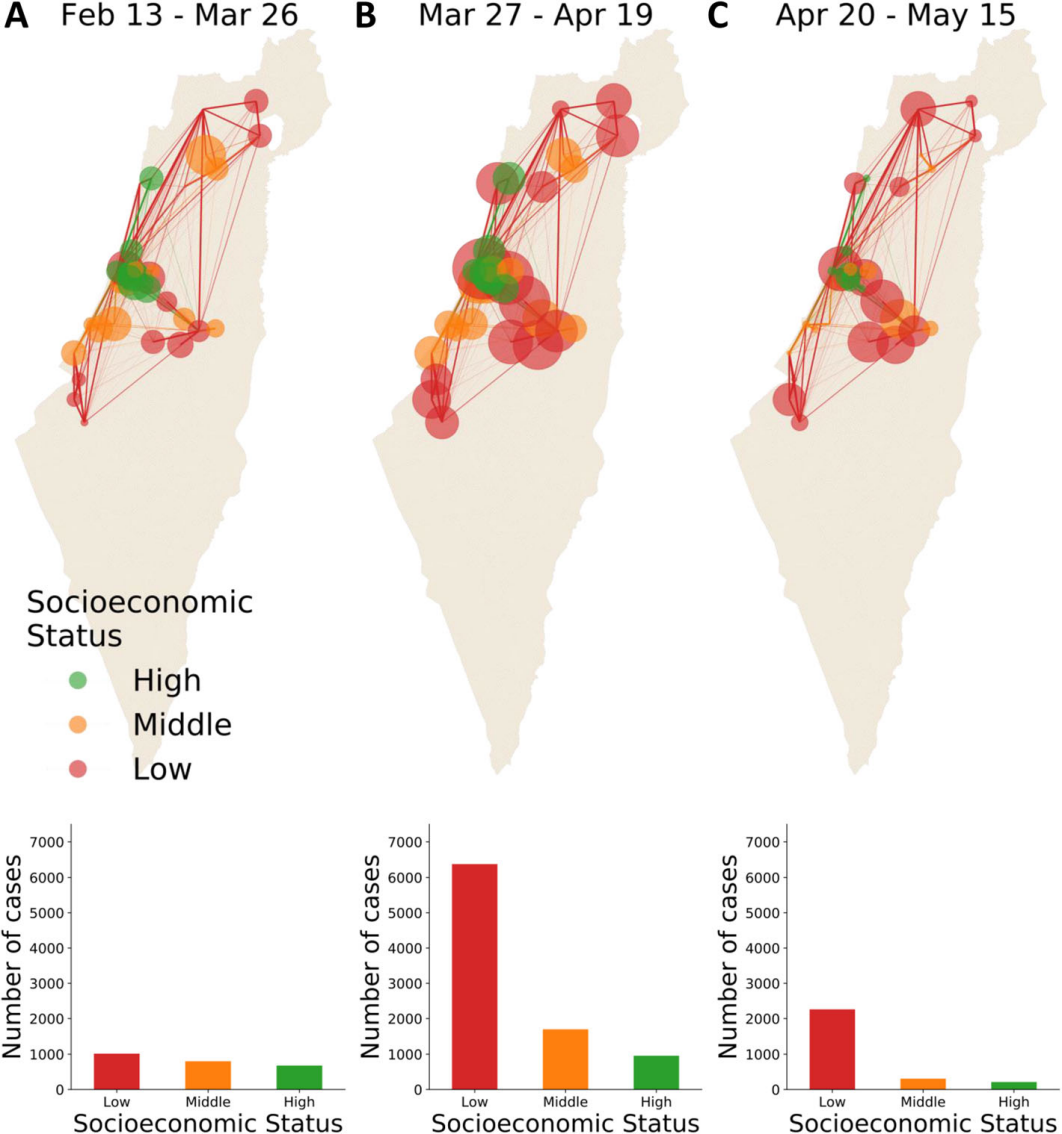
Association between mobility and poverty in COVID-19 transmission. Spatiotemporal transmission by socioeconomic status. We present the 50 municipalities with the highest incidence. Each circle represents one municipality. The radius (presented on a logarithmic scale for clarity) reflects the total number of new cases reported during the corresponding period. The colors reflect socioeconomic status. The lines between the municipalities represent the traffic of each municipality, wherein the line thickness represents the relative traffic intensity and the color matches the color of the SES of origin. We present below each map the number of reported cases among different SEGs for three periods corresponding to (**a**) the early phase before restrictions started, (**b**) from the time of restrictions and until the restrictions were lifted, and (**c**) after restrictions were lifted

To analyze the relationship among poverty, mobility, and transmission (Fig. 2), we divided the data into three periods: 13 Feb-26 Mar, 27 Mar-19 Apr, and 20 Apr-15 May, corresponding to 1) the early phase before restrictions started, 2) the time from restrictions until they were first lifted, and 3) after the restrictions were lifted. For each period, we ranked municipalities with a population of > 10,000 residents based on the number of new cases per person observed in each period. For improved clarity of Fig. 2, we present the 50 most prevalent municipalities. We calculated for each city the number of newly reported cases, the SES, and the distribution of travels to the other 49 municipalities.

### Transmission model

**Fig. 3:**
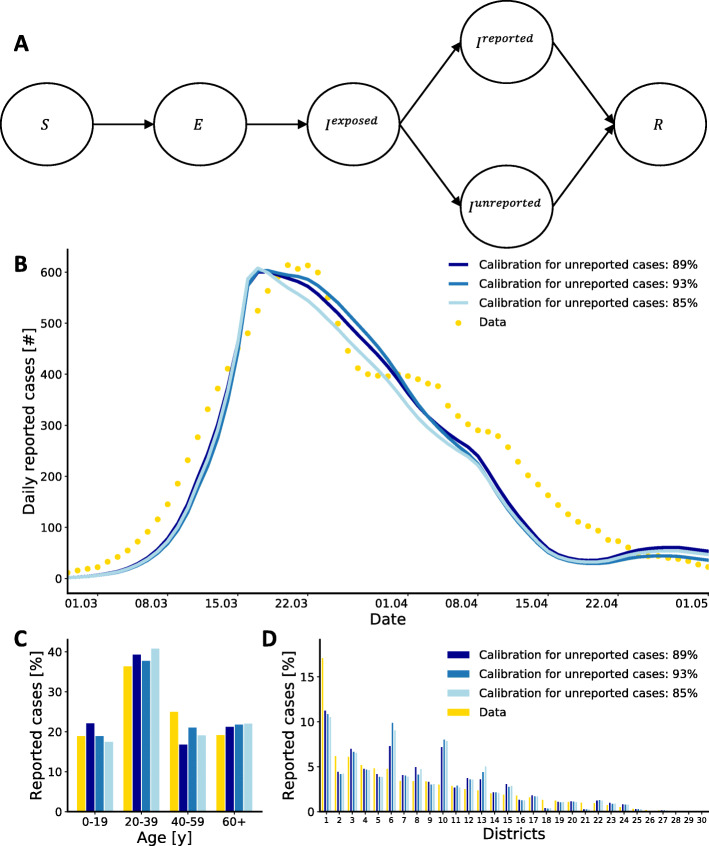
Structure and fit of the transmission model. **a** Compartmental diagram of the transmission model. Susceptible individuals *S* transition to the exposed compartment with a force of infection λ, where they are infected but not yet infectious, until moving to an early infectious compartment at rate *σ*, in which they do not show symptoms but may transmit. Infected individuals in the early stage move to a reported *I*^*Reported*^ or unreported *I*^*Unreported*^ infectious period, in which they may have a mild or an asymptomatic infection until death or complete recovery. For clarity of depiction, age, risk, and region stratifications are not displayed. **b** Time series of reported daily COVID-19 cases and model fit countrywide. **c** Data and model fit to the age distribution among COVID-19 infections. **d** Data and model fit to the 30 subdistricts covering Israel

We developed a dynamic model for age-, risk- and region-stratified SARS-CoV-2 infection progression and transmission in Israel. Our model is a modified susceptible exposed infected recovered (SEIR) compartmental framework [20], whereby the population is stratified into health-related compartments, and transitions between the compartments change over time (Fig. 3a). To model age-dependent transmission, we stratified the population into age groups: 0–4 years, 5–9 years, 10–19 years, 20–29 years, 30–39 years, 40–49 years, 50–59 years, 60–69 years and ≥ 70 years. We distinguished high-risk and low-risk individuals in each age group based on the ACIP case definition [21, 22]. We also distinguished the 250 regions covering Israel in the model.

The mean incubation period of SARS-CoV-2 is 6.4 days (95% CI, 5.6 to 7.7 days) [23, 24], but early evidence shows that viral shedding occurs during a presymptomatic stage [25, 26]. Thus, we considered an exposure period *E* and an early infectious period *I*^*exposed*^. Underreporting arises from asymptomatic cases or mild cases in individuals who do not seek care. Thus, following the early infectious phase, individuals in the model transition either to an infectious and reported compartment *I*^*reported*^ or to an infectious and unreported compartment *I*^*unreported*^ [27, 28].

Multiple infections with SARS-CoV-2 are not yet fully understood. A recent study indicated that there is protective immunity following infection [29]. This result is consistent with a previous study indicating that for SARS-CoV-1, memory T cells persist for up to 11 years [30]. In addition, similar to other respiratory infections, it is likely that if reinfection occurs, it is less severe and less transmissive [31]. Thus, we assumed that upon recovery, individuals are fully protected, which is consistent with other SARS-CoV-2 transmission models [32] (Additional file 1: Supplementary information). Altogether, our model includes 5 ∗ 9 ∗ 2 ∗ 250 = 22,500 compartments (*health* − *compartments* ∗ *age* − *groups* ∗ *risk* − *groups* ∗ *regions*).

### Force of infection and seasonality

The rate at which individuals transmit depends on (i) contact mixing patterns between the infected individual and his or her contact, (ii) age-specific susceptibility to infection, (iii) region-based behavioral susceptibility, and (iv) potential seasonal forcing.

Age-specific contact rates were parameterized using data from an extensive survey of daily contacts [33] and data from CBS regarding the household size in each region. In addition, we utilized the aggregate mobility data regarding movement patterns within and between 250 regions as observed in the data during routine and following restrictions (Additional file 1: Supplementary information). We specifically distinguished the contact patterns of infected individuals for different locations, namely, at home, at work and during leisure, such that the number of contacts was based on the extensive survey [33] and the household size, whereas the mixing patterns were based on the locations of the individuals as analyzed using the mobile data. These contact data reveal frequent mixing between similar age-groups, moderate mixing between children and people their parents’ age, and infrequent mixing among other groups. The data based on mobility reveal more frequent mixing between individuals of similar SES, at similar geographical distances, and with cultural similarities (Additional file 1: Supplementary information).

We distinguished between in-home and out-of-home transmission. We evaluated the in-home transmission is independent of age and, based on a previous retrospective study, that suggested a value of 0.16 for the household attack rate [19]. The age-specific susceptibility rate for out-of-home individuals *β*_*j*_ was parameterized by calibrating our model with daily COVID-19 records.

To account for behavioral susceptibility, we explicitly considered in our model a parameter reflecting the order to maintain physical distancing, *κ*_*p*_. The high regional variations in susceptibility were parameterized based on fertility rates and socioeconomic characteristics. The fertility rate in Israel correlates with population density, household size, and SES. Specifically, we computed for each region the relative change in mobility compared to routine. Our analysis indicated that for regions of low SES, the change was lower, which was reflected in our model by higher susceptibility (Additional file 1: Supplementary information). The use of regional fertility and relative change in mobility allowed us to refrain from calibrating the model to an excessive number of unknown parameters and avoid overfitting.

Seasonal patterns have been observed in common circulating human coronaviruses (HCoVs), mostly causing infections in humans between December and May in the Northern Hemisphere [34]. The two HCoVs 229 E and OC43 show distinct winter seasonality. In addition, many coronaviruses in animals exhibit a distinct seasonal pattern of incidence in their natural hosts [35]. There is growing evidence that SARS-CoV-2 is also seasonal, with the optimal setting for transmission in Israel occurring during winter [36]. Thus, we considered in our base-case seasonal forcing by including general seasonal variation in the susceptibility rate of the model as

$$ T(t)=\left(1+\mathit{\cos}\left(\frac{2\pi \left(t+\varphi \right)}{365}\right)\right), $$

in which *φ* is the seasonal offset. This formulation was previously shown to capture the seasonal variations in several respiratory infections, including RSV and influenza [31, 37]. We incorporated the possible values of *φ* to reflect peaks from December through February (Additional file 1: Supplementary information).

### Model calibration

To empirically estimate unknown epidemiological parameters (Additional file 1: Table S5), we calibrated our model to daily age-stratified cases of COVID-19 confirmed by PCR tests in 30 subdistricts covering Israel. The calibration was conducted on a 30-subdistrict level rather than in the 250 regions to ensure that there were sufficient time-series data points in each location for each age-group. The data were reported by the Israeli Ministry of Health between February and May and included daily information for the patients, including age, residential zone, underlying conditions, and clinical outcomes, including hospitalizations and death. To calibrate the model, we minimized the mean squared error (which is also the maximum likelihood estimation assuming the error is normally distributed) between the model projections of reported cases and the daily COVID-19-confirmed cases data.

Due to the uncertainty regarding the proportion of unreported cases, we calibrated our model to different scenarios. Specifically, underreporting is affected by testing policy and testing capabilities for each country, as well as individuals’ tendency to seek care once clinical symptoms appear. Additionally, underreporting is affected by the severity of the infection, which is associated with age [18]. Thus, we chose different age-specific estimates for the proportion of underreporting, ranging from 5.5–14 unreported cases for a single reported case. These estimates are based on observations from screenings conducted in unpublished data from Israel and are consistent with data from Denmark, Czechia, Netherlands; Santa Clara, California [14, 18, 38] (Additional file 1: Table S1). Due to the uncertainty related to positive predictive values of serological screenings, we also tested a scenario of two unreported cases for a single reported case to confirm the robustness of our findings.

To account for the age variation, we considered the detailed serological data from Santa Clara [14]. We also calibrated our model with scenarios assuming different phases of seasonal peaking between December 21 and February 21, as well as scenarios with no seasonality. The final transmission model included five parameters without constraints imposed from previous data: reduced susceptibility due to physical distancing *κ*_*p*_ and susceptibility rate based on age groups *j*: 0–19, 20–39, 40–59, and > 60 (Additional file 1: Supplementary information).

### Model simulations

We evaluated the effectiveness of temporal lockdown strategies in reducing morbidity and mortality by simulating the model for 1 year and 3 years or until disease elimination. Each strategy considered includes a threshold for activation of a lockdown, and the groups considered for lockdown were as follows: 1) the entire population in the region, 2) daycare- and school-age children between 0 and 19 years of age (children), 3) high-risk groups and individuals > 65 years of age (elderly). Specifically, to model the lockdown strategies, we defined an indicator for each region as the weekly number of new-reported cases per 10,000 people. Each week, we examined whether the indicator exceeds a certain threshold for each region. If so, a lockdown was activated for the following week. This process was continued for 1–3 years.

We simulated the lockdowns in our model based on the mobility patterns we observed between March 26 and April 16 during which a stay-home orders were applied. In this period, school and daycare centers were closed, and for non-essential workplace only 10% of employees from private and public sectors were allowed to work. Individuals were required to stay in a radius of 100 m from their home except for grocery and health-related shopping. We define the local lockdowns as a stay home order for individuals living in that specific region and not to those who travel into the region.

We projected the number of individuals who will die under each strategy by utilizing detailed information from the Israeli Ministry of Health (Additional file 1: Table S2). Specifically, we calculated for each age- and risk-group the proportion of individuals who died out of the reported cases. We multiplied these proportions with the daily model projections of newly reported cases and summed this product to calculate the total projected number of deaths. We also accounted for the uncertainty regarding the estimated probabilities. We define the efficiency of a lockdown strategy as the total number of deaths averted per total lockdown days. The number of deaths averted is calculated as the projected number of deaths with no lockdowns minus the number of deaths projected when the considered strategy is applied. The total lockdown days are calculated as the projected duration of lockdown multiplied by the population’s size affected by the lockdown.

This approach is in accordance with the standard mechanism of cost-effectiveness analysis. Specifically, lockdowns negatively impact individuals’ Quality Adjusted Life Years (QALY) by increasing mental and physical health burden [39, 40]. In addition, solely from the losses in productivity, 1 week of national lockdown is equivalent to ~ 35,000 years of loss by World Health Organization (WHO) criteria for a cost-effective intervention [4, 41]. Thus, we counted the total days of lockdowns (i.e., number of individuals under lockdowns multiplied by the lockdown duration) as they correlate with the lockdowns’ health and economic burden.

## Results

### Human mobility and poverty

We utilized aggregated and anonymized information about mobility based on cellular data. The data specifies movement patterns of > 3 million users within and between 2630 zones covering Israel, on an hourly basis, from February 1, 2020, to May 16, 2020. This period corresponds to the period from a month before the COVID-19 outbreak began in Israel until 16,600 cases were reported. Each zone includes ~ 3500 residents with available information regarding several socioeconomic characteristics, including household size, age distribution, mean socioeconomic score, and religion.

During the aforementioned period, the government applied and lifted several movement restrictions. We define a mobility index (MI) as the daily proportion of individuals who traveled > 1.5 km away from their home. While a sharp decline in cases has been observed in the overall population following restrictions, the decline varied considerably among individuals of different SESs. Specifically, during routine days, the low-SES population had the lowest MI. Shortly after the restrictions started, this trend changed, and populations of all SESs had similar MIs, while during the lockdowns, the high-SES population had the lowest MI (Fig. 1a).

Before the COVID-19 outbreak, the population was highly clustered such that people of a specific SES typically traveled to zones where the residents matched their SES and were, therefore, more likely to meet with each other (Fig. 1b; Additional file 1: Figs. S1 and S2). Likewise, people of similar demographic groups, such as those with the same religious affiliations, typically traveled to zones where the residents matched their group. These trends further intensified following the restrictions (Fig. 1c). Notably, the clustering was not attributable to only the geographical distance, as many high-SES zones are geographically close to the low-SES zone.

### Human mobility and poverty explain transmission

To explore the spatiotemporal effect of human mobility and poverty on transmission, we calculated the number of new cases and the amount of travel between zones observed during three periods: February 13–March 26, March 27–April 20, and April 20–May 20 (Fig. 2). These periods correspond to 1) the early phase before restrictions started, 2) between the time of restrictions and until the restrictions were lifted, and 3) after restrictions were lifted. Our analysis indicated that during the first period, the infection was evenly distributed among different SESs. During the second period, 71% of the cases were residents of zones with a low SES, particularly religious orthodox Jews. During the third period, 81% of the cases were residents of low SES, mainly residents of zones of Israeli Arabs and orthodox Jewish people. We also identified a high correlation ranging from 79.2–82.8% (*p* value< 0.001) with a lag of 12–14 days between the MI and the disease growth factor, i.e., the number of new cases daily per active case (Additional file 1: Fig. S3). This lag includes the incubation period, the time from symptom onset until a test is conducted, and the time until the test results arrive.

We integrated the daily mobility data into an age-, region-, and risk-stratified model for SARS-CoV-2 transmission. Model parameters were calibrated to the number of new cases daily in 30 subdistricts covering Israel. With only five free parameters, the model recapitulated SARS-CoV-2 trends (Fig. 3). For example, the calibrated model showed that the national SARS-CoV-2 infections peaked during March 17–25 (Fig. 3b) and yielded age and regional distributions of SARS-CoV-2 consistent with the data (Fig. 3c and d). Our calibration further indicated that a model ignoring mobility poorly captured the spatiotemporal dynamics and provided an overestimation of disease transmission (Additional file 1: Table S5). Specifically, our tests suggested that models that do not include the mobility data (*p*.value< 0.01) and the regional fertilities (*p*.value< 0.01) were significantly worse. We also found that a model that accounted for seasonal forcing yielded a higher, but not significant (*p* value< 0.35), likelihood than a model that did not account for seasonal forcing (Additional file 1: Table S5).

### Focused lockdowns reduce morbidity and mortality

**Fig. 4:**
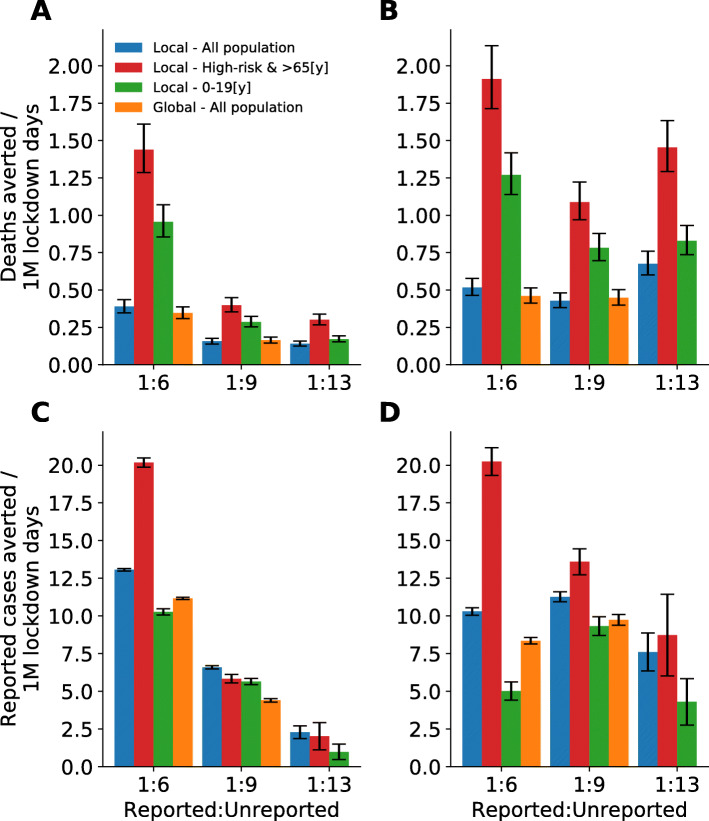
Efficiency of lockdown strategies. Median and interquartile values of the projected number of (**a**, **b**) deaths and (**c**, **d**) reported cases averted per 1 million lockdown days due to the implementation of lockdown strategies (**a**, **c**) after one year and (**b**, **d**) after three years. The threshold for lockdowns in a local region is 5/10,000 [reported cases/individuals]

As transmission varied considerably among regions, we projected the number of the total reported cases and deaths for 1–3 years under local and temporal lockdown strategies. Specifically, we simulated three strategies triggered by a threshold of daily COVID-19 incidence in each of the 250 regions. We evaluated the efficiency of the lockdown strategies, defined as the number of reported cases or deaths averted per lockdown day (Fig. 4; Additional file 1: Fig. S5). We found that the local strategy of targeting the elderly was substantially more efficient than the national strategy. For example, assuming one reported case for every six unreported cases (i.e.,1:6 [reported:unreported]), and a lockdown threshold of 5/10,000 (reported cases/individuals), a strategy targeting the elderly is 3.3–5.1 times more efficient than a global strategy in terms of death, and 1.79–1.82 in terms of reported cases after 1 year (Fig. 4a and b). Moreover, the number of days under local lockdown varies considerably between regions, demonstrating the importance of differential lockdowns (Additional file 1: Tables S6, S7, and S8).

**Fig. 5:**
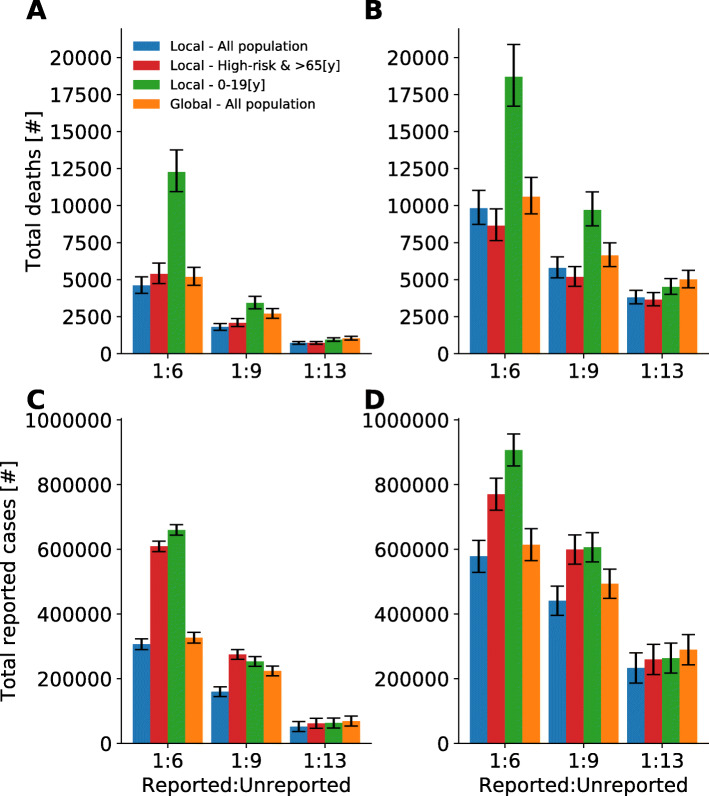
Effectiveness of lockdown strategies. Median and interquartile values of the projected number of deaths, (**a**, **b**), and reported cases, (**c**, **d**) after implementing strategies following one year, (**a**, **c**), and three years, (**b**, **d**), from implementation. The threshold for lockdowns in a local region is 5/10,000 [reported cases/individuals]

We evaluated the effectiveness of each strategy in reducing morbidity and mortality (Fig. 5; Additional file 1: Fig. S6). We found that a strategy locally targeting the elderly yielded a lower number of deaths than a strategy targeting children. For example, assuming one reported case for every six unreported cases (i.e.,1:6 [reported:unreported]) and a lockdown threshold of 5/10,000 (reported cases/individuals), a strategy targeting the high-risk group resulted in 4736–6122 deaths, while only targeting children resulted in 10,935–13,765 deaths after 1 year (Fig. 5a). In addition, for lockdown thresholds exceeding 5/10,000 (reported cases/individuals), which aligns with Israel’s current practice, a strategy locally targeting the elderly either is projected to be the most effective or is comparable to the most effective strategies in reducing mortality. Although comparable on the effectiveness level, such a policy includes 2.2–5.5 times fewer individuals under lockdowns (Additional file 1: Fig. S4). In terms of cases, we found that a local strategy targeting all age groups is the most effective. For example, for the same transmission settings and lockdown threshold, a local strategy targeting all age groups resulted in 290,155–323,022 reported cases after 1 year (Fig. 5c). These trends were consistent across vast ranges of epidemiological parameters, different plausible ranges of threshold values, and different seasonal forcing considerations.

## Discussion

Our key findings suggest that COVID-19 infection does not spread uniformly in the population, and thus, intervention strategies should focus primarily on protecting elderly and individuals with underlying conditions in regions of outbreaks. Such a strategy can reduce mortality while enabling daily routine for a vast majority of the population.

Our results indicate that a local strategy targeting all age groups is the most effective to reduce reported cases, while a local strategy targeting individuals at high-risk is the most efficient. The reason behind this finding is that due to the lower contact mixing, the contribution of a recovered elderly on decreasing transmission is lower than the contribution of a non-elderly recovered [42].

Our work demonstrates that to understand the spatiotemporal dynamics of transmission, models must account for mobility as well as behavioral aspects that are associated with sociodemographic and socioeconomic factors. In particular, we found that SARS-CoV-2 is more likely to spread in more impoverished regions and is affected by human mobility. The intensive interactions likely led to higher transmission in developed countries than in developing countries. However, our model suggested that people of low SES are at higher risk due to poorer compliance and larger household size. Thus, to contain the COVID-19 outbreak, more resources should be devoted to helping impoverished regions.

Our results show that mobility patterns highly correlates with disease growth factor. This observation is also in line with a previous study [43], which underscored the importance of utilizing cellular data to predict and control the ongoing pandemic. However, there are several limitations to the use of mobility data [44–46]. Cellphones’ mobility data may not capture protective behavior such as wearing a mask and maintaining physical distancing. This observation is crucial to exploring transmission dynamics as it may vary considerably across subpopulations and change drastically during the pandemic. Thus, we denote that our study identifies and relies on correlations and associations and does not attempt to assume causality.

Our analyses indicate that localized lockdowns with incidence thresholds as low as five reported cases in 10,000 individuals are essential to decrease mortality. This finding underscores the importance of maintaining a high level of testing [47], particularly in regions with elevated risk of transmission. However, with such a strategy, at least 2500 total years of lockdowns (equivalent to a one-day lockdown of 912,500 individuals) are required to prevent a single death. Considering that 1 day of lockdown is equivalent to a quality of life value that is ~ 0.85 times that in a routine day [48], even local lockdowns should be prudently considered from a health economic perspective. Thus, future modeling studies should also include localized and temporal massive screening efforts, which result in more focused quarantines and isolations than massive control measures.

As in any modeling study, we made several assumptions. Nationwide and local lockdowns are powerful, yet heavy, control measures. Thus, the local strategies tested in our model should be applied only if containment cannot be achieved via less drastic measures to the economy such as the use of contact tracing to break the chains of infection, requiring the use face masks and educating to maintain physical distancing. We denote that these measures were applied in Israel and were taken into consideration in our model indirectly by our calibration process. Thus, our model suggests the disease cannot be contained by these measures in the extent they were implemented.

We assumed in our model that there is a long-lasting protective immunity following infection which is consistent with previous human coronavirus types [29, 30, 32]. However, a recent study suggested that people are unlikely to produce long-lasting protective antibodies against this virus [49]. If, indeed, a rapid waning is possible, this highlights the importance to protect the elderly in regions of high outbreaks.

Social contact patterns are a crucial factor in the transmission of COVID-19 and other infectious agents in a population [31, 50]. We did not consider changes in the contact patterns due to reported illness, including self-isolation. The MOH’s epidemiological investigations suggested that 67% of the reported infections occurred within the household, and the duration between infection and the COVID-19 test result is, on average, 10.5 days. Additionally, due to considerably low testing, most cases are unreported. Thus, the effect of self-isolation was minimal and was not explicitly modeled. This observation is in line with a recent study indicating that the majority of incidences may be attributable to transmission from a combination of the presymptomatic stage and asymptomatic infections [51]. Moreover, effective contact tracing and isolation of individuals on symptoms onset may prevent the need for lockdowns [52, 53]. Future studies can evaluate the effect of these measures together with local and global lockdowns to effectively reduce morbidity and mortality.

Our local lockdowns correspond to regions with a population of ~ 36,000 people. A smaller lockdown may be more efficient but could not be tested by our model. Moreover, it might be hard to enforce a true ‘local lockdown’ as people would travel for non-necessities outside their region. This travel outside could lead to potentially a quicker dissemination of the disease. However, from a policy perspective, our definition for local lockdown makes it more feasible. Additionally, with the growing evidence of a disproportionate risk from COVID-19 to the elderly [18, 54], focused control measures are likely to be conducted in retirement homes and facilities with populated communities at high risk, which we did not explicitly account for in our model [55]. Although the transmission dynamics are unlikely to change with such focused interventions, the overall mortality is expected to be lower than what we have found.

While there is a debate in the literature regarding the extent of infectiousness and transmissibility in children [56], our results highlighted a not less important question: to whom do children transmit? Our findings reveal that children are less likely to transmit to populations at risk, and thus, a differential lockdown strategy that targets children may be even harmful.

## Conclusion

We showed that using aggregated and anonymized human mobility data from cellular phones under the General Data Protection Regulation (GDPR) guidelines is a powerful tool to improve the understanding of transmission dynamics and to evaluate the effectiveness of control measures. Our transmission model predicted that rather than nationwide lockdowns, applying temporal and localized lockdowns that focus on elderly can substantially reduce mortality. Such focused measures will enable a vast majority of the population to maintain a daily routine.

## Additional File

**Additional file 1:.** Supplementary information for: Human mobility and poverty as key drivers of COVID-19 transmission and control.pdf

## Data Availability

The medical data that support the findings of this study are publicly available by the Israeli Ministry of Health, https://data.gov.il/dataset/covid-19. The aggregated and anonymized human mobility data which were used under the license for the current study are not publicly available. Data, however, available from the authors upon reasonable request.

## Abbreviations

MI: Mobility index
GDPR: General data protection regulation
SES: Socioeconomic status
CBS: Central bureau of statistics
QALY: Quality-adjusted life-year
WHO: World health Organization
