# Supplementary material for: Human mobility and poverty as key drivers of COVID-19 transmission and control
Source: BMC Public Health. 2021 Mar 25;21:596. doi: 10.1186/s12889-021-10561-x (PMC7993906; doi:10.1186/s12889-021-10561-x)
Supplement: Supplementary file 1 — Additional file 1:. Supplementary information for: Human mobility and poverty as key drivers of COVID-19 transmission and control.pdf [file 12889_2021_10561_MOESM1_ESM.docx]

**Supplementary information for:** **Human mobility and poverty as key drivers of COVID-19 transmission and control**

Matan Yechezkel^1,¶^, Amit Weiss^1,¶^, Idan Rejwan^1,¶^, Edan Shahmoon^1^, Shachaf Ben-Gal^1^, Dan Yamin^1,2,*^

^1^ Laboratory for Epidemic Modeling and Analysis, Department of Industrial Engineering, Faculty of Engineering, Tel Aviv University, Tel Aviv 6997801, Israel

^2^ Center for Combatting Pandemics, Tel Aviv University, Tel Aviv 6997801, Israel

¶ These authors contributed equally to this work

* To whom correspondence should be sent: Dan Yamin, PhD Email: [dan.yamin@gmail.com](mailto:dan.yamin@gmail.com)

**Table of Content**

[1. **Model** 2](#_Toc42628893)

[1.1. The model 2](#_Toc42628894)

[1.2. Model transitioning 4](#_Toc42628895)

[1.3. Force of infection 5](#_Toc42628896)

[2. **Fixed parameters** 8](#_Toc42628897)

[2.1. Contact mixing patterns 8](#_Toc42628898)

[2.2. Relative reduction in travels 13](#_Toc42628899)

[2.3. Epidemiological parameters 15](#_Toc42628900)

[3. **Calibrated parameters** 20](#_Toc42628901)

[4. **Further simulation results** 26](#_Toc42628902)

[5. **References** 30](#_Toc42628903)

## **Model**

### **The model**

We developed a dynamic model for age-, risk- and regions-stratified SARS-Cov-2 infection progression and transmission in Israel. Our model is a modified Susceptible-Exposed-Infected-Recovered (SEIR) compartmental framework [1], whereby the population is stratified into health-related compartments, and transitions between the compartments occurs over time (Main text, Fig. 3). To model age-dependent transmission, we stratified the population into nine age groups: 0–4 years, 5-9 years, 10-19 years, 20-29 years, 30-39 years, 40-49 years, 50-59 years, 60-69 years and ≥70 years. [2–4]. We distinguished between high-risk and low-risk individuals for each age group based on the ACIP case definition [5,6]. We also distinguish in the model between 250 regions covering Israel.

Multiple infections with SARS-Cov-2 is yet fully understood. A recent study indicated that there is a protective immunity following infection in humans [7] and animals [8]. This result is in-line with a previous study indicating that for SARS-Cov-1, Memory T cells persist for up to 11 years [9]. In addition, similarly to other respiratory infections, it is likely that if re-infection occurs, it is less severe and less transmissive [10]. Thus, we assumed that upon recovery individuals are fully protected for the entire season wich consistent with other SARS-COV-2[11,12].

The mean incubation period of SARS-Cov-2 is 6.4 days (95% CI, 5.6 to 7.7 days) [13,14], but first evidence shows viral shedding occurs during a pre-symptomatic stage [15,16]. Thus, we considered an exposed period $E$, and an early infectious period $I^{exposed}$. Underreporting arises from asymptomatic cases or mild cases of individuals that do not seek care [17–20]. Thus, following the early infectious phase, individuals in the model transition either to an infectious and reported compartment $I^{reported}$, or to infectious and unreported compartment $I^{unreported}$.

To enable in our model for a subset of the population to go for intervention (e.g., 30% of the individuals from specific regions, age groups or risk-group to go under lockdown during a selected time period), we also specifically distinguish between those who undergo and those who did not undergo an intervention.

Accordingly, we stratified the population into six health-related compartments: susceptible$S_{j,k,r,q}(t)$, exposed but not yet infectious$E_{j,k,r,q}(t)$, infectious at early stage$I_{j,k,r,q}^{exposed}(t)$, reported infectious$I_{j,k,r,q}^{reported}(t)$, unreported infectious $I_{j,k,r,q}^{unreported}(t)$ and recovered $R_{j,k,r,q}(t)$, such that at any given time t (in days) the population is fixed and scaled to one. Namely,

|  | $\sum_{j} \sum_{k} \sum_{r} \sum_{q} \left[ S_{j,k,r,q}\left( t \right)+ E_{j,k,r,q}\left( t \right)+ I_{j,k,r,q}^{exposed}(t)+ I_{j,k,r,q}^{reported}(t)+I_{j,k,r,q}^{unreported}(t)+R_{j,k,r,q}(t) \right]= \sum_{j} \sum_{k} \sum_{r} \sum_{q} N_{j,k,r,q} =1,$ | (1) |
| --- | --- | --- |

where the index $j\in\{0-4y,5-10y,\ldots,>70y\}$ represents the age-group of each individual, index $k\in\left\{ 1,2,\ldots,250 \right\}$ specifies the home region of each individual, index $r\in\{L,H\}$ specifies the risk-group of each individual (i.e. High-risk, or low-risk) and index $q\in\{intervention, non-intervention\}$ represent the intervention-group of each individual.

### **Model transitioning**

Susceptible individuals $S_{j,k,r,q}\left( 0 \right)$, transition to the exposed compartment $E_{j,k,r,q}(t)$, with force of infection $\lambda_{j,k,q}(t)$, depending on their age-group *j* home region-group *k* and their intervention-group *q*. At this compartment individuals are infected but not yet infectious until they move at rate $\sigma$ to an infectious compartment $I_{j,k,r,q}^{exposed}(t)$ , where they are at the early stage of the infectious period. Infected individuals at early stage of their infectious period, then move at rate $\delta$ to the late infectious period, where they can become to a unreported case (having non to mild symptoms) with probability $f_{j,r}$ which results in transition to $I_{j,k,r,q}^{unreported}(t)$. With probability of $(1-f_{j,r})$ they can become to a reported case (having moderate to severe symptoms), which results in transition to $I_{j,k,r,q}^{reported}(t)$. After infectious period, individuals’ transition into the recovered compartment at rate $\gamma$, $R_{j,k,r,q}(t)$,. (See Section, 2.3 Epidemiological parameters). We also consider a function of the initial spreaders with time $\varepsilon_{j,k,r}(t)$, that reflects the individuals exposed to the virus the entered Israel from overseas between February 21 2020 - and March 9, 2020. Thus, the transmission model is composed of the following system of difference equations:

|  | $S_{j,k,r,q}\left( t \right)=S_{j,k,r,q}\left( t-1 \right)-\lambda_{j,k,q}\left( t \right)\cdot S_{j,k}\left( t-1 \right)$*,*  $E_{j,k,r,q}\left( t \right)=E_{j,k,r,q}\left( t-1 \right)+\lambda_{j,k,q}\left( t \right)\cdot S_{j,k}\left( t-1 \right)-\sigma\cdot E_{j,k,r,q}\left( t-1 \right)+\varepsilon_{j,k,rq}(t),$  $I_{j,k,r,q}^{exposed}\left( t \right)=I_{j,k,r,q}^{exposed}\left( t-1 \right)+\sigma\cdot E_{j,k,r,q}\left( t-1 \right)-\delta\cdot I_{j,k,r,q}^{exposed}\left( t-1 \right),$  $I_{j,k,r,q}^{reported}\left( t \right)= I_{j,k,r,q}^{reported}\left( t-1 \right)+\left( 1-f_{j,r} \right)\delta\cdot I_{j,k,r,q}^{exposed}\left( t-1 \right)-\gamma\cdot I_{j,k,r,q}^{reported}\left( t-1 \right),$  $I_{j,k,r,q}^{unreported}\left( t \right)= I_{j,k,r,q}^{unreported}\left( t-1 \right)+f_{j,r}\delta\cdot I_{j,k,r,q}^{exposed}\left( t-1 \right)-\gamma\cdot I_{j,k,r,q}^{unreported}\left( t-1 \right),$  $R_{j,k,r,q}\left( t \right)= R_{j,k,r,q}\left( t-1 \right)+\gamma\cdot\left( I_{j,k,r,q}^{reported}\left( t-1 \right)+I_{j,k,r,q}^{unreported}\left( t-1 \right) \right)$*,*  with initial conditions:  $S_{j,k,r,q}\left( 0 \right)= N_{j,k,r,q}.$  $E_{j,k,r,q}\left( 0 \right)=I_{j,k,r,q}^{exposed}\left( 0 \right)=I_{j,k,r,q}^{reported}\left( 0 \right)=I_{j,k,r,q}^{unreported}\left( 0 \right)=R_{j,k,r,q}\left( 0 \right)=0$*.* | (2) |
| --- | --- | --- |

### **Force of infection**

The rate at which individuals transmit SARS-Cov-2 at time t is $\lambda_{j,r,q}(t)$. This rate depends on the combination of (i) contact mixing patterns between an infected individual and his or her contacts, (ii) age-specific susceptibility to infection, (iii) region-based behavioral susceptibility, and (iv) a potential seasonal forcing.

We incorporate age- and region-specific contact patterns between individuals, represented by contact rate between an infected individual in age-group $i$, region-group $l$ and each of their contacts with susceptible in age-group $j$,region-group $k$, for different locations: at home, at work and during leisure, for each day $t$denoted by$C_{\left( l,i \right),\left( k,j \right)}^{\tau}(t)$, such that i$\tau\in\{Home,Work,Leisure\}$, is the location index of the contact location index. The contact matrix $C_{\left( l,i \right),\left( k,j \right)}^{\tau}(t)$ is detailed in section 2.1 **Contact mixing patterns.**

We distinguish between in-home versus out-of-home transmission. Consistent with a previous study [21], we assume the in-home transmission to be fixed and independent of age, $\beta_{Home}$. (See Section 2.3 Epidemiological parameters). To account for the reduced probability of infection in house following a recovery of other house members, we multiple the susceptibility inside household, $\beta_{Home}$, by decay function $\psi_{k}\left( t \right)=\frac{S_{k}\left( t-1 \right)}{S_{k}(0)}.$ This function serve as an unbiased estimator to the proportion of susceptible individuals in the house Age-specific susceptibility rate for individuals out-of-home $\beta_{j}$, was parameterized by calibrating our model with daily COVID-19 records (See Section 3. calibrated parameters).

To account for behavioral susceptibility, we explicitly considered in our model a parameter reflecting the order to maintain physical distancing, $\kappa_{p}$, as vast number of countries, including Israel, adopted measures such as physical-distancing to control the susceptibility of SARS-CoV-2 [22]. This parameter was calibrated to the epidemiological data of COVID-19 in Israel. Moreover, the high regional variations in susceptibility were parameterized based on fertility rates and socioeconomic characteristics relative to the national average, using the data from Central Bureau of Statistics (CBS), $\alpha_{k}$. Specifically, we computed for each region the relative reduction in travels >1.5 km compared to routine $M_{j,k,q}$(See Section 2.2 Relative reduction in travels). Our analysis indicated that for regions of low SES the change was lower, which was reflected by our model with higher susceptibility.

Seasonal patterns have been observed in common circulating HCoVs, mostly causing infections in humans between December and May in the Northern Hemisphere [23]. The two human coronaviruses 229 E and OC43 show distinct winter seasonality. In addition, many coronaviruses in animals do exhibit a distinct seasonal pattern of incidence in their natural hosts [24]. There is growing evidence that SARS-CoV-2 is also seasonal, with the optimal setting for transmission in Israel during winter [25,26]. Thus, we considered in our base-case seasonal forcing by including general seasonal variation in the susceptibility rate of the model as

|  | $T\left( t \right)=1+\cos\left( \frac{2\pi(t+\phi)}{365} \right).$ | (3) |
| --- | --- | --- |

in which $\varphi$is seasonal offset. This formulation was previously shown to capture the seasonal variations of several respiratory infections including RSV and influenza [10,27]. We incorporated possible values of $\varphi$ to reflect peak from December thru February (See Section 2.3 Epidemiological parameters).

Taken together, the force of infection $\lambda_{j,k,q}(t)$ is given by

|  | $\lambda_{j,k,q}\left( t \right)= M_{j,k,q}\cdot\kappa_{p}\cdot T\left( t \right)\cdot\left( \beta_{home}\cdot\psi_{k}\left( t \right)\cdot\sum_{i} \sum_{l} \sum_{p} C_{\left( l,i \right),\left( k,j \right)}^{Home}(t)\sum_{r} \left( I_{j,k,r,p}^{exposed}\left( t-1 \right)+I_{j,k,r,p}^{reported}\left( t-1 \right)+I_{j,k,r,p}^{unreported}\left( t-1 \right) \right)+\beta_{j}\cdot\alpha_{k}\cdot\left[ \sum_{i} \sum_{l} \sum_{p} \sum_{\tau\in\left\{ Work,Leisure \right\}} C_{\left( l,i \right),\left( k,j \right)}^{\tau}(t)\sum_{r} \left( I_{j,k,r,p}^{exposed}\left( t-1 \right)+I_{j,k,r,p}^{reported}\left( t-1 \right)+I_{j,k,r,p}^{unreported}\left( t-1 \right) \right) \right] \right)$ | (4) |
| --- | --- | --- |

## **Fixed parameters**

### **Contact mixing patterns**

At the core of the transmission model lies the contact mixing patterns between a susceptible individual and infectious individual$C_{\left( l,i \right),\left( r,j \right)}^{\tau}(t)$. Similar to a previous study [21], the contact matrices depends on the age-group and region of residency for the susceptible individual $(l,i$), the age group and region of residency for an infectious individual $\left( r,j \right)$at location $\tau\in\{Home,Work,Leisure\}$ on day $t$ . Here we detail the process of how we conducted the contact-mixing.

***Household contacts***

We estimated the contact mixing at home for each region based on the average household size and its age distribution from the Israeli Central Bureau of Statistics (CBS) [19,28]. We assume all individuals in the same household will meet with each other daily regardless of the control measures applied by the country (e.g. lockdowns). The CBS data suggest that low socioeconomic status is characterized by larger and younger household size. ‬‬‬‬‬‬‬‬‬‬‬‬‬‬‬‬‬‬‬‬‬‬‬‬‬‬‬‬‬‬‬‬‬‬‬‬‬‬‬‬‬‬‬‬‬‬‬‬‬‬‬‬‬‬‬‬‬‬‬‬‬‬‬‬‬‬‬‬‬‬‬‬‬‬‬‬‬‬‬‬‬‬‬‬‬‬‬‬‬‬‬‬‬‬‬‬‬‬‬‬‬‬‬‬‬‬‬‬

***Work and leisure contact patterns***

*Age-specific contacts*

We parametrized the age-specific contact rates using data from a survey of daily contacts collected in eight European countries [29]. This contact data includes contact rates for different locations: works (or school for children <10), leisure. In addition, the data exhibits frequent mixing between similar age-groups, moderate mixing between children and adults in their thirties (likely their parents), and infrequent mixing between other groups. To generate the age-specific contact mixing used in our model, we used the means of each age-group over the eight countries. To ensure the matrices is symmetric and convert between age-groups used in the survey to those used in out model, we adjusted the contact matrices according to the means for reciprocal age group pairing [10].

*Origin-destination from mobility data*

Our data includes mobility records based on cellular data of >3 million users from one of the largest telecommunication companies in Israel. The data specifies movement patterns within and between 2,630 zones covering Israel, on an hourly basis, from February 1, 2020, and until May 16, 2020. To ensure privacy, if in a given hour less than 50 individuals are identified in the zone, the number of reported individuals is set to zero. We determined the location of individuals based on the triangulation of cell towers, which was found accurate to 300 meters in most cases but varied to 1 km in less populated areas. We defined users as residents of a zone based on location in which they had the highest number of signals on most nights during February 2020.

We used this data to develop aggregated origin-destination (OD) matrices between and within zones. To refrain from signal noises and identify stay points, we track only locations where users stayed for at least 15 minutes within a distance threshold of 1.7 km. The OD matrices serve as a proxy to the flow from each region to another.

Next, we integrated data from the Central Bureau of Statistics (CBS) that specifies for each zone several socioeconomic characteristics, including population size, household size, age distribution, socioeconomic score, and dominant religion. Each zone includes ~3,500 residents. For each zone, we scaled the number of resident users of the telecommunication company to match with the actual number of residents in the zone, as recorded by the Israeli CBS. Grouping the zones by SES, and scaling for each zone the daily number of travels to one, we created an origin-destination traveling probability matrix. We found that the population is clustered, such that people of specific SES are more likely to travel to zones of the same SES during routine and even more likely during movement restrictions. These findings remain consistent when partitioning the population into resolution of 10 socioeconomic clusters, comprising the different SESs. Additionally, a similar phenomenon is observed when partitioning the population by Religious Affiliations to Arab, orthodox and non-orthodox Jewish, and also for the combination of both religious affiliation and socioeconomic clusters (Figs. S1 and S2).


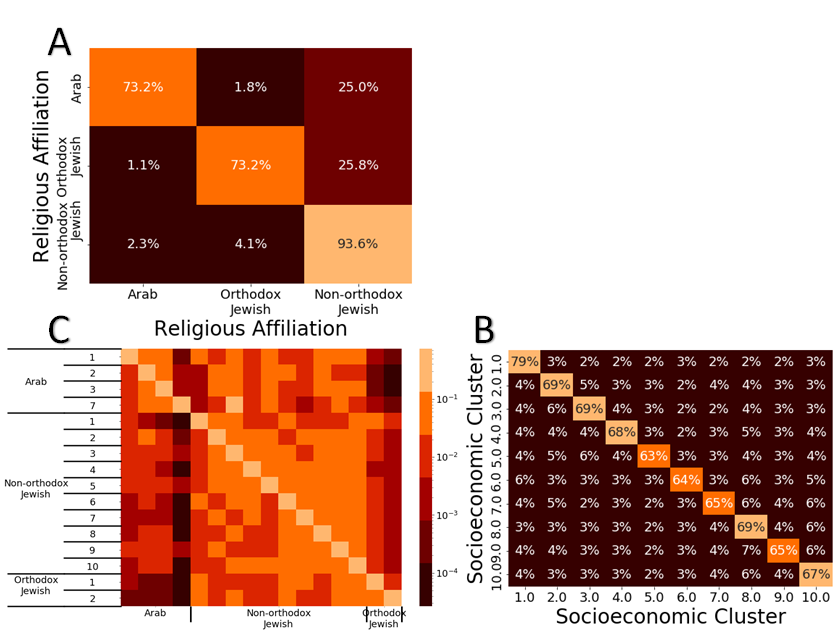


**Fig. S1.** **Traveling patterns during routine.** Traveling patterns during February 2-29 based on (A) religious affiliation, (B) socioeconomic status, and (C) religious affiliation and socioeconomic status.


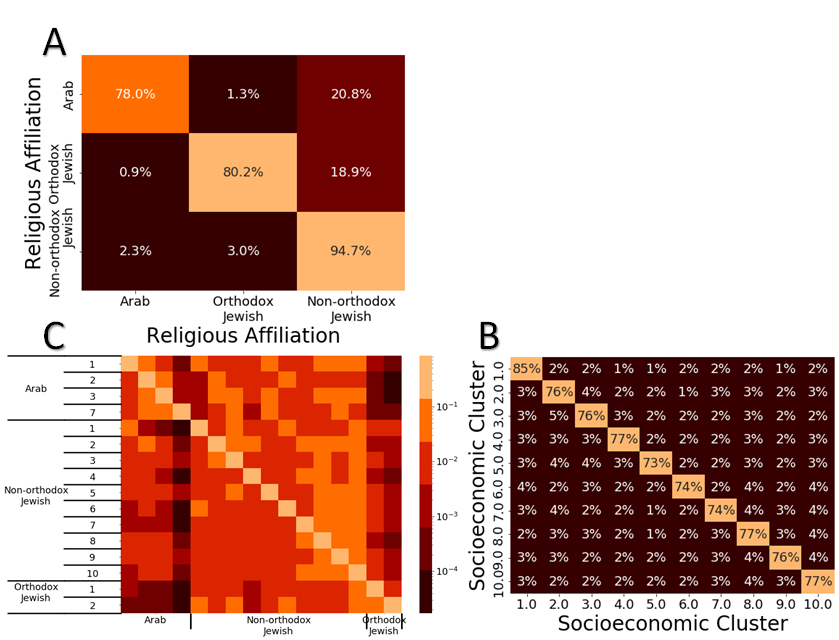

**Fig. S2. Traveling patterns during COVID-19 outbreak**. Traveling patterns during March 26-April 18 based on (A) religious affiliation, (B) socioeconomic status, and (C) religious affiliation and socioeconomic status.

We used this data to develop two aggregated origin-destination (OD) matrices between and within regions from during work time 08:01-17:00 and leisure time 17:01-23:00. To incorporate the time depended travels following restrictions periods and routine we developed the two OD for the following periods: February 21 – March 13, March 14 – March 16, March 17 – March 25, March 26 – April 2, April 3 – April 6, April 7 – April 16, April 17 – May 4, May 5 – May 11.

To integrate the age-specific contact matrices and the OD matrices we multiplied the number of contacts for each age-group by the travel distribution for each region in the OD matrices. We assumed that at work, children at the age of 0-9 years old, remains at their home region. We also assumed that at leisure time children at the age of 0-9 years old movement patterns are like their parents.

### **Relative reduction in travels**

For each region, we computed the relative reduction in travels >1.5 km $M_{j,k,q}$. This measure was done scaling the daily proportion of travels more than 1.5 km out-of-home.

|  | $M_{j,k,q}=\frac{MI_{q}\left( t \right)-\min_{t} \left( MI_{q}\left( t \right) \right)}{\max_{t} \left( MI_{q}\left( t \right) \right)-\min_{t} \left( MI_{q}\left( t \right) \right)}$ | (5) |
| --- | --- | --- |

To compute this minimal and maximal values and refrain from outliers, we averaged the three minimal and three maximal values. This measure was found to be highly correlative with disease growth factor ranging between 79.2-82.8% (p value<0.001) for a shift of 12-14 days (Fig. S3). Thus, we incorporated for each region this measure in the model.


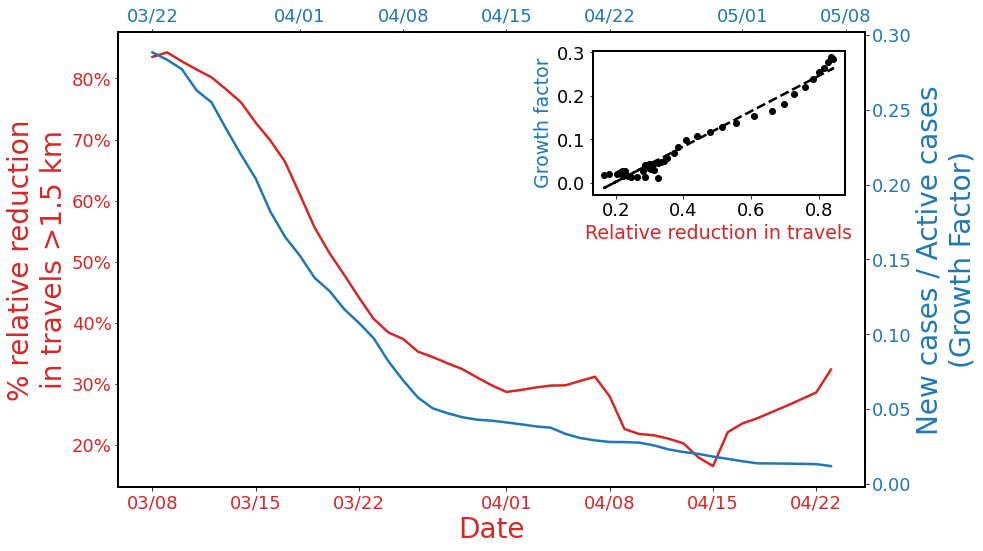


**Fig. S3**. **Mobility ahead of transmission**. Percentage relative reduction in travels from home between March 8 and April 22 (red) and new cases per active cases between March 22 and May 8 (blue). Both plots show the weekly average. The correlation between the two is 97.0% (inserted graph).

### **Epidemiological parameters**

*Unreported cases*

Under reporting arises from asymptomatic cases or mild cases of individuals that do not seek for care. The severity of SARS-Cov-2 infection is associated with age- and risk- group [30]. In addition, underreporting is affected by testing policy and testing-capabilities for each country, as well as the tendency of individuals to seek for care once clinical symptoms appear. PCR or serological screenings have yet to be conducted in Israel. Thus, we evaluated unreported cases based on PCR and serological screenings from the Czech Republic, Denmark, and Santa Clara, California, and Iceland. Similarly, to Israel, as to May 14^th^, 2020 these countries are characterized with high rates of testing and low number of severe cases. In addition, hospitals were not overwhelmed. Serological screenings from the Czech Republic suggested that each reported case corresponds to ~5.5 unreported cases [19,20], whereas estimates from Santa Clara suggested at least 14 unreported cases for each single reported case [17]. Taken together we chose to present estimates of unreported ratios 1:5.5 (Scenario A), 1:9 (Scenario B), and 1:14 (Scenario C). It is not clear how much neutralizing antibodies are sufficient to ensure protection, and thus it is possible serological screenings serve as over estimation to determine exposure. Thus, to determine the robustness of our findings, we also considered an extreme scenario of 1:2 (Scenario D).

We estimated the proportion of under reporting for each age-group by scaling the estimates from Santa-Clara Study to the age reported cases in this region [31]. This analysis suggested that younger age-groups are more likely to be unreported. Conservatively, we assumed that all cases among individuals at high-risk are reported. Using these estimates and based on the reported cases in Israel between February 20^th^ - May 14^th^ ,2020, we obtained that overall proportion of unreported cases is 85% for scenario A, 89% for scenario B, 93% for scenario C and 69% for scenario D.

**Table S1.** **proportion of unreported cases.** proportion of unreported cases among individuals at high risk and low risk stratified by age and overall reported cases based on the reported cases observed in Israel between February 20 and May 14, 2020.

| Scenario | Risk \ Age | 0-19 | | 20-64 | ≥65 | |
| --- | --- | --- | --- | --- | --- | --- |
| A | **Low** | 0.97 | | 0.85 | 0.68 | |
|  | **High** | 0.97 | | 0.85 | 0.68 | |
|  | **Total** | 0.85 | | | | |
| B | **Low** | 0.95 | | 0.89 | 0.80 | |
|  | **High** | 0.95 | | 0.89 | 0.80 | |
|  | **Total** | 0.89 | | | | |
| C | **Low** | 0.99 | | 0.93 | 0.84 | |
|  | **High** | 0.99 | | 0.93 | 0.84 | |
|  | **Total** | 0.93 | | | | |
| D | **Low** | 0.92 | 0.67 | | | 0.43 |
|  | **High** | 0.92 | 0.67 | | | 0.43 |
|  | **Total** | 0.69 | | | | |

*Case fatality*

The probability of death for each age-and risk-group given a reported case was evaluated based on the Israeli Ministry of Health case report data (Table S2).

**Table S2**. **Probability of death for each age-and risk-group given a reported case.** i.e., case fatality rate (CFR)

| Age-group | Risk-group | Base-case value | Distribution |
| --- | --- | --- | --- |
| 0-19 | **High** | 0.001% | $Beta(4,241662)$ |
| 20-59 | **High** | 0.89% | $Beta(4,410)$ |
| 60-69 | **High** | 1.48% | $Beta(5,312)$ |
| $\boldsymbol{\geq70}$ | **High** | 12.03% | $Beta(52,378)$ |
| 0-19 | **Low** | 0.001% | $Beta(4,241662)$ |
| 20-59 | **Low** | 0.06% | $Beta(5,7759)$ |
| 60-69 | **Low** | 1.06% | $Beta(11,995)$ |
| $\boldsymbol{\geq70}$ | **Low** | 11.33% | $Beta(95,741)$ |

*Initial morbidity(aboard)*

The initial morbidity in Israel was imported by 491 citizens who returned from overseas. The first infected traveler identified on February 20, and by March 9^th^ ,2020 a self-quarantine was mandatory for all returning. Most of the flights to Israel arrive from the developed countries. Thus, we distributed the these cases in each day of the 18 days proportionally to the daily new cases in Italy, which had the hardest hit among developed countries [32]. To account for under reporting, we multiplied the number of cases in each day according to the unreported scenarios we considered (Table S1). We entered these initial spreaders, $\varepsilon_{j,k,r,i}(t)$, to the exposed compartment.

*Susceptibility at-home*

We distinguish between in-home versus out-of-home transmission. Consistent with a previous study [21]. We specifically distinguish between the susceptibility of those settings. We estimated the in-home susceptibility rate, $\beta_{home}$ , based on a previous study that showed a secondary attack rate of 16.3% throughout the entire infectious period [33].

**Table S3**. **Fixed parameters used in the transmission model.**

| Parameter | Description | Value | Reference |
| --- | --- | --- | --- |
| $\boldsymbol{N}_{\boldsymbol{j,k,r,q}}$ | Population size of risk-group r age-group j in region k | Varies between regions | [5] |
| $\frac{\boldsymbol{1}}{\boldsymbol{\sigma}}$ | Mean duration of exposed period | $4.1 days$ | [13–16] |
| $\frac{\boldsymbol{1}}{\boldsymbol{\delta}}$ | Mean duration of early infectious period | $2.3 days$ | [13–16,34,35] |
| $\boldsymbol{f}_{\boldsymbol{j,r}}$ | Unreported probabilities | Table S1 | [17,18,30] |
| $\boldsymbol{\varphi}$ | Seasonal phase | December 21 ($\varphi=60$),  January 21 ($\varphi=29$),  February 21 ($\varphi=0$). | [12,24–26,36] |
| $\frac{\boldsymbol{1}}{\boldsymbol{\gamma}}$ | Mean duration of late infectious period (in reported and unreported cases) | $7 days$ | [15] |
| $\boldsymbol{C}_{\left( \boldsymbol{l,i} \right)\boldsymbol{,}\left( \boldsymbol{r,j} \right)}^{\boldsymbol{\tau}}\boldsymbol{(t)}$ | Contact rate between an infected individual in age-group $i$, region-group $l$ and each of their contacts with susceptible in age-group $j$,region-group $k$, for different location $\tau$, for each day $t$. |  | [2,19,28,29]‬‬‬‬‬‬‬‬‬‬‬‬‬‬‬‬‬‬‬‬‬‬‬‬‬‬‬‬‬‬‬‬‬‬‬‬‬‬‬‬‬‬‬‬‬‬‬‬‬‬‬‬‬‬‬‬‬‬‬‬‬‬‬‬‬‬‬‬‬‬‬‬‬‬‬‬‬‬‬‬‬‬‬‬‬‬‬‬‬‬‬‬‬‬‬‬‬‬‬‬‬‬‬‬‬‬‬‬ |
| $\boldsymbol{\alpha}_{\boldsymbol{k}}$ | Fertility rate for each region *k* relative to the nation’s mean. |  | [19,37,38]‬‬‬‬‬‬‬‬‬‬‬‬‬‬‬‬‬‬‬‬‬‬‬‬‬‬‬‬‬‬‬‬‬‬‬‬‬‬‬‬‬‬‬‬‬‬‬‬‬‬‬‬‬‬‬‬‬‬‬‬‬‬‬‬‬‬‬‬‬‬‬‬‬‬‬‬‬‬‬‬‬‬‬‬‬‬‬‬‬‬‬‬‬‬‬‬‬‬‬‬‬‬‬‬‬‬‬‬ |
| $\boldsymbol{\rho}_{\boldsymbol{j,r}}$ | Probability of death for each age-and risk-group given a reported case | Table S2 | [39] |
| $\boldsymbol{\beta}_{\boldsymbol{home}}$ | In-home susceptibility rate | 0.018 | [33] |

## **Calibrated parameters**

To estimate empirically unknown epidemiological parameters, we calibrated our model to daily age-stratified cases of COVID-19 confirmed by PCR tests in 30 subdistricts covering Israel between March 1 until May 10. We shifted the data 11 days backward, to compensate for the lag between the date of infection and the date of first positive SARS-CoV2 test result, which was found to be 10.5 days on average according to MOH’s epidemiological investigations. We applied a central moving average with window of three days before and after the data point, on the data to reduce noise caused by weekly patterns.

The calibration was conducted on a 30-subdistrict level rather than 250 regions to ensure there are sufficient time-series data points in each location for each age group. The stratification is based on the 16 formal districts, which we further stratified such that the sub districts will be homogenous in terms of their SES and religious affiliation (Table S4). To calibrate the model to the incidence data, we maximized the likelihood assuming a normal distribution of the error between model predictions and incidence data. This was achieved by using the truncated Newton (TNC) algorithm. We calibrated the model for 16 different scenarios of unreported cases and seasonal forcing. The final transmission model included five parameters without constraints imposed from previous data: reduced susceptibility due to physical distancing $\kappa_{p}$, and susceptibility rate based on age-groups *j*: 0-19, 20-39, 40-59, and >60 (Table S5).

We used an F-test of equality of variances to compare between models 1) with vs. without consideration of seasonal forcing, 2) with and without consideration of human mobility, 3) with and without consideration of regional fertility. We denote that in all three comparisons, the number of calibrated parameters is constant and equal to five. Our tests suggested that models that do not include the mobility data (p.value<0.01), and the regional fertilities (p.value<0.01) were significantly worse. We also found that models that accounted for seasonal forcing yielded higher, but not significant (p value<0.35), likelihood than models that did not account for the seasonal forcing.

**Table S4**. **30 subdistricts calibrated.**

| Sub-district number | Name | Population Size |
| --- | --- | --- |
| 1 | Jerusalem and sub. | 778,503 |
| 2 | Bet Shemesh | 120,164 |
| 3 | Jerusalem and sub. (Orthodox Jewish) | 265,313 |
| 4 | Zefat | 138,618 |
| 5 | Zefat (Israeli Arabs) | 23,772 |
| 6 | Kinneret (Jewish) | 98,178 |
| 7 | Jezreel Valley (Israeli Arabs) | 159,112 |
| 8 | Jezreel Valley (Jewish) | 351,446 |
| 9 | Akko (Israeli Arabs) | 357,341 |
| 10 | Akko (Jewish) | 314,607 |
| 11 | Ramat Hagolan | 51,980 |
| 12 | Haifa (Israeli Arabs) | 35,637 |
| 13 | Haifa (Jewish) | 589,951 |
| 14 | Hadera (Israeli Arabs) | 115,000 |
| 15 | Hadera (Jewish) | 315,593 |
| 16 | Sharon (Israeli Arabs) | 85,729 |
| 17 | Sharon (Jewish) | 412,638 |
| 18 | Petah Tiqwa (Israeli Arabs) | 27,455 |
| 19 | Petah Tiqwa (Orthodox Jewish) | 49,549 |
| 20 | Petah Tiqwa (Secular Jewish) | 680,836 |
| 21 | Ramla | 323,352 |
| 22 | Rehovot | 661,079 |
| 23 | Tel Aviv – Yafo | 820,271 |
| 24 | Bnei Brak | 211,259 |
| 25 | Tel Aviv suburbs | 464,974 |
| 26 | Ashqelon | 559,556 |
| 27 | Beer Sheva (Israeli Arabs) | 196,311 |
| 28 | Beer Sheva (Jewish) | 504,831 |
| 29 | Judea and Samaria | 267,832 |
| 30 | Judea and Samaria (Orthodox Jewish) | 155,095 |

**Table S5**. **Calibrated parameters.**

| Model configuration | Seasonal forcing peak | Unreported [%] | Physical distancing Coefficient$\boldsymbol{\kappa}_{\boldsymbol{physical}}$ | Susceptibility among age-group 0-19[y]  $\boldsymbol{\beta}_{\boldsymbol{0-19}}$ | Susceptibility among age-group 20-39[y]  $\boldsymbol{\beta}_{\boldsymbol{20-39}}$ | Susceptibility among age-group 40-59[y]  $\boldsymbol{\beta}_{\boldsymbol{40-59}}$ | Susceptibility among age-group 60+[y]  $\boldsymbol{\beta}_{\boldsymbol{60+}}$ | Likelihood of calibration to data  $\boldsymbol{-}\log\boldsymbol{(l)}$ |
| --- | --- | --- | --- | --- | --- | --- | --- | --- |
| Full model | **No-seasonality** | **69** | 0.248 | 0.094 | 0.054 | 0.042 | 0.311 | -25.766 |
| Full model | **No-seasonality** | **85** | 0.232 | 0.119 | 0.053 | 0.052 | 0.166 | -25.743 |
| Full model | **No-seasonality** | **89** | 0.234 | 0.057 | 0.076 | 0.047 | 0.116 | -25.494 |
| Full model | **No-seasonality** | **93** | 0.246 | 0.119 | 0.036 | 0.054 | 0.184 | -25.876 |
| Full model | **December 21** | **69** | 0.272 | 0.038 | 0.023 | 0.020 | 0.128 | -25.856 |
| Full model | **December 21** | **85** | 0.306 | 0.044 | 0.021 | 0.024 | 0.109 | -25.862 |
| Full model | **December 21** | **89** | 0.355 | 0.025 | 0.021 | 0.025 | 0.144 | -25.998 |
| Full model | **December 21** | **93** | 0.274 | 0.058 | 0.015 | 0.023 | 0.083 | -25.917 |
| Full model | **January 21** | **69** | 0.364 | 0.043 | 0.025 | 0.024 | 0.151 | -25.835 |
| Full model | **January 21** | **85** | 0.310 | 0.050 | 0.025 | 0.028 | 0.123 | -25.876 |
| Full model | **January 21** | **89** | 0.390 | 0.032 | 0.029 | 0.028 | 0.159 | -25.975 |
| Full model | **January 21** | **93** | 0.322 | 0.066 | 0.018 | 0.027 | 0.097 | -25.901 |
| Full model | **February 21** | **69** | 0.347 | 0.063 | 0.039 | 0.033 | 0.248 | -25.822 |
| Full model | **February 21** | **85** | 0.464 | 0.051 | 0.036 | 0.034 | 0.199 | -25.813 |
| Full model | **February 21** | **89** | 0.417 | 0.052 | 0.045 | 0.041 | 0.229 | -25.916 |
| Full model | **February 21** | **93** | 0.411 | 0.100 | 0.030 | 0.034 | 0.157 | -25.827 |
| Without mobility | **January 21** | **85** | 0.127 | 0.022 | 0.035 | 0.022 | 0.162 | -25.129 |
| Without mobility | **January 21** | **89** | 0.133 | 0.031 | 0.029 | 0.022 | 0.133 | -25.206 |
| Without mobility | **January 21** | **93** | 0.098 | 0.049 | 0.030 | 0.023 | 0.121 | -25.139 |
| Without fertility | **January 21** | **85** | 0.633 | 0.056 | 0.027 | 0.018 | 0.013 | -25.311 |

## **Further simulation results**

We found that a global lockdown strategy had a larger temporal effect than local lockdowns and had by greater oscillations (Fig. S4). We present here a model with seasonal forcing. Our model projections suggested that global lockdowns were less efficient and effective compared to a strategy that targets locally the elderly. However, due to high variability between the 250 regions considered, some regions undergo multiple lockdowns, while others will not undergo lockdowns. Local lockdowns that specifically target children decreases the local morbidity, but in the long run increases mortality, while lockdowns of individuals at high-risk has a moderate impact on transmission but decreases mortality.

These findings where robust across all settings considered (Tables S3 and S5), when we accounted for seasonal forcing (Main text, Figs. 4 and 5), and without seasonal forcing (Fig. S5).


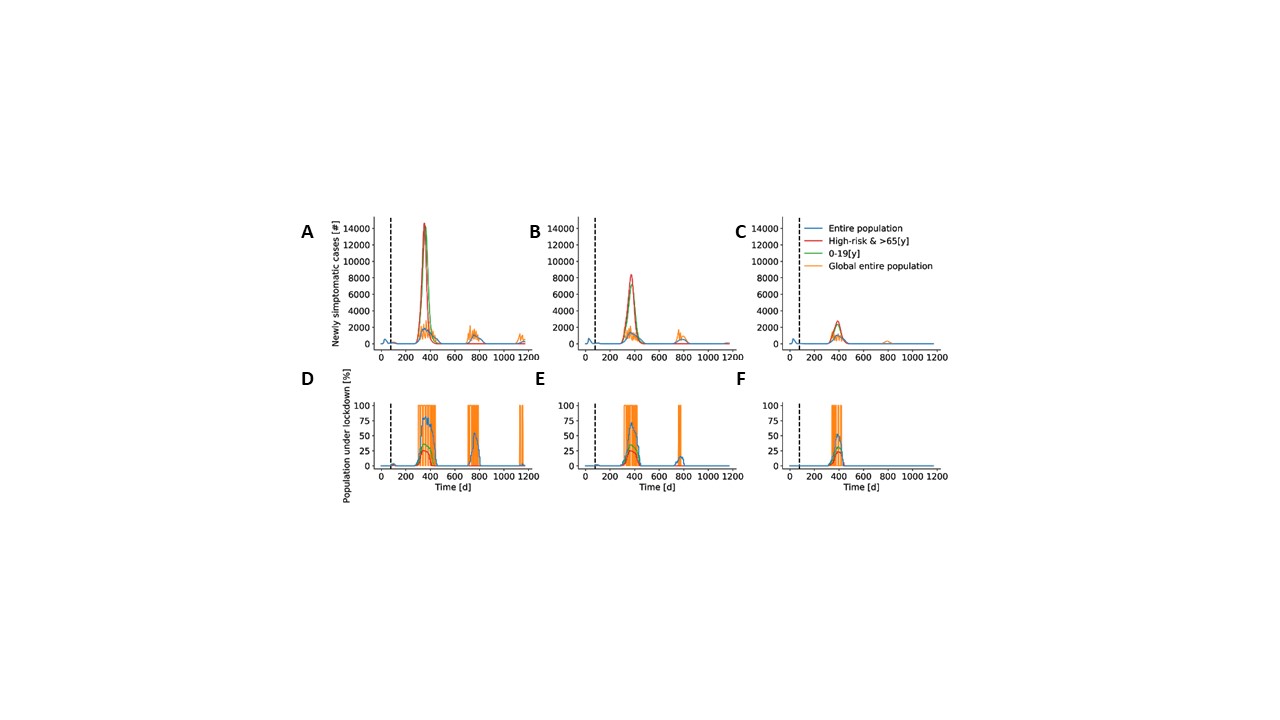


**Fig. S4. Model demonstration for a** **threshold of 1 per 10000 for the lockdown strategies with seasonal forcing peaking on January 21.** (A – C) projected daily new reported cases for different lockdown strategies. (D – F) Projected daily percentage of population under lockdown. (A, D) for a unreported cases of 85%. (B, E) for 89%, and (C, F) for 93%.

| 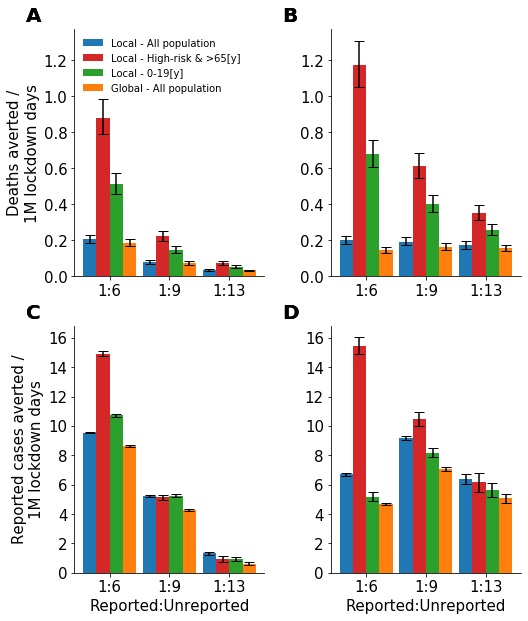 |
| --- |
| **Fig. S5. Efficiency of lockdown strategies.** Median and interquartile values of the projected number of (A, B) deaths and (C, D) reported cases averted per 1 million lockdown days due to the implementation of lockdown strategies (A, C) after one year and (B, D) after three years. The threshold for lockdowns in a local region is 1/10,000 [reported cases/individuals]. |

| 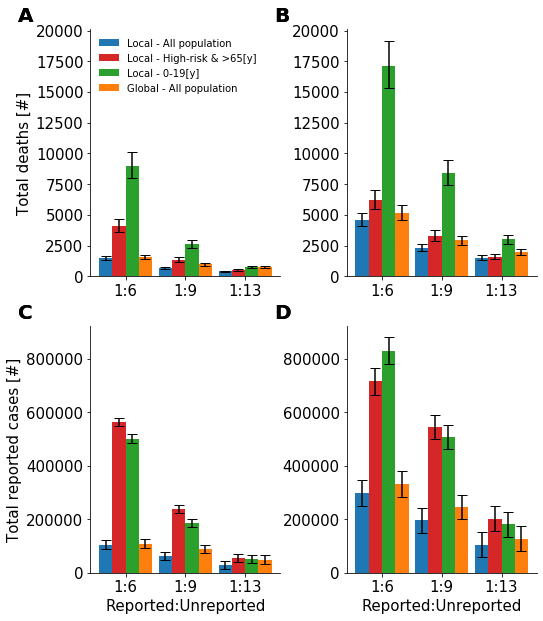 |
| --- |
| **Fig. S6. Effectiveness of lockdown strategies.** Median and interquartile values of the projected number of (A, B) deaths and (C, D) reported cases after implementation of strategies (A, C) after one year and (B, D) after three years. The threshold for lockdowns in a local region is 1/10,000 [reported cases/individuals]. |


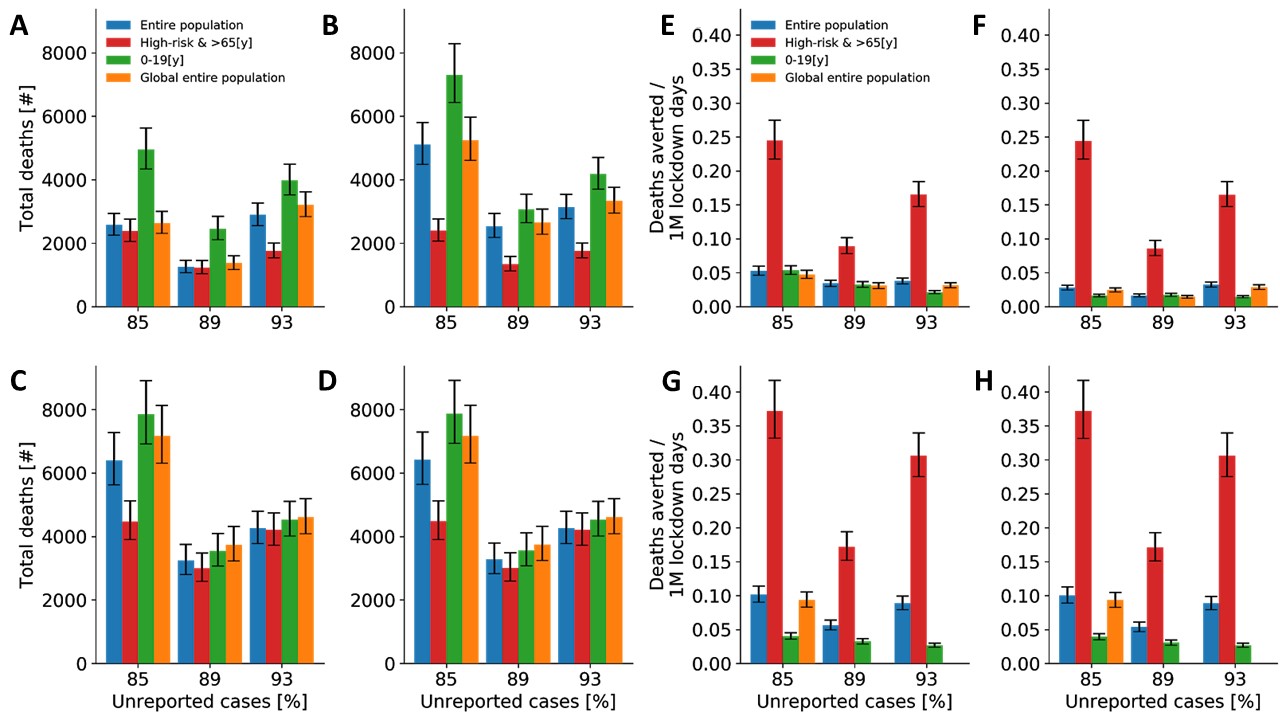


**Fig. S7. Effectiveness and efficiency of temporal-local lockdowns without seasonal forcing.** Median and interquartile values of model projections after implementation of strategies (A, C, E, G) after one year and (B, D, F, H) after three years. (A, B, E, F) The thresholds for lockdowns in a local region are 1/10,000 [cases/individuals] and (C, D, G, H) 5/10000 [cases/individuals]. Effectiveness (A – D), efficiency (E – G).

**Table S6. The total number of days under local lockdown**. The number of regions for each local lockdown day range and strategy during the three-years horizon. The thresholds for lockdowns in a local region are 5/10,000 [cases/individuals]. The proportion of unreported cases is 85% (i.e., 1:6 [reported:unreported cases]).

|  | Number of regions | | |
| --- | --- | --- | --- |
| Policy | **Kids** | **Risk** | **All** |
| Total number of days under local lockdown: |  |  |  |
| 0-20 days | 0 | 0 | 1 |
| 21-40 days | 14 | 15 | 32 |
| 41-60 days | 216 | 222 | 198 |
| > 60 days | 11 | 4 | 10 |

**Table S7. The total number of days under local lockdown**. The number of regions for each local lockdown day range and strategy during the three-years horizon. The thresholds for lockdowns in a local region are 5/10,000 [cases/individuals]. The proportion of unreported cases is 89% (i.e., 1:9 [reported:unreported cases]).

|  | Number of regions | | |
| --- | --- | --- | --- |
| Policy | **Kids** | **Risk** | **All** |
| Total number of days under local lockdown: |  |  |  |
| 0-20 days | 1 | 1 | 3 |
| 21-40 days | 17 | 9 | 213 |
| 41-60 days | 223 | 230 | 25 |
| > 60 days | 0 | 1 | 0 |

**Table S8. The total number of days under local lockdown**. The number of regions for each local lockdown day range and strategy during the three-years horizon. The thresholds for lockdowns in a local region are 5/10,000 [cases/individuals]. The proportion of unreported cases is 93% (i.e., 1:13 [reported:unreported cases]).

|  | Number of regions | | |
| --- | --- | --- | --- |
| Policy | **Kids** | **Risk** | **All** |
| Total number of days under local lockdown: |  |  |  |
| 0-20 days | 102 | 141 | 229 |
| 21-40 days | 138 | 100 | 12 |
| 41-60 days | 1 | 0 | 0 |
| > 60 days | 0 | 0 | 0 |

## **References**

1. Vynnycky E, White R. Introduction. The basics: infections, transmission and models. An Introduction to Infectious Disease Modelling. 2010.

2. Medlock J, Galvani AP. Optimizing influenza vaccine distribution. Science (80- ). 2009. doi:10.1126/science.1175570

3. Ndeffo Mbah ML, Medlock J, Meyers LA, Galvani AP, Townsend JP. Optimal targeting of seasonal influenza vaccination toward younger ages is robust to parameter uncertainty. Vaccine. 2013. doi:10.1016/j.vaccine.2013.04.052

4. Yamin D, Gavious A, Solnik E, Davidovitch N, Balicer RD, Galvani AP, et al. An Innovative Influenza Vaccination Policy: Targeting Last Season’s Patients. Koelle K, editor. PLoS Comput Biol. 2014;10: e1003643. doi:10.1371/journal.pcbi.1003643

5. Molinari NAM, Ortega-Sanchez IR, Messonnier ML, Thompson WW, Wortley PM, Weintraub E, et al. The annual impact of seasonal influenza in the US: Measuring disease burden and costs. Vaccine. 2007. doi:10.1016/j.vaccine.2007.03.046

6. Fiore AE, Fry A, Shay D, Gubareva L, Bresee JS, Uyeki TM, et al. Antiviral agents for the treatment and chemoprophylaxis of influenza --- recommendations of the Advisory Committee on Immunization Practices (ACIP). MMWR Surveill Summ Morb Mortal Wkly report Surveill Summ / CDC. 2011.

7. Ni L, Ye F, Cheng M-L, Feng Y, Deng Y-Q, Zhao H, et al. Detection of SARS-CoV-2-specific humoral and cellular immunity in COVID-19 convalescent individuals. Immunity. 2020 [cited 13 May 2020]. doi:10.1016/j.immuni.2020.04.023

8. Bao L, Deng W, Gao H, Xiao C, Liu J, Xue J, et al. Reinfection could not occur in SARS-CoV-2 infected rhesus macaques. bioRxiv. 2020; 2020.03.13.990226. doi:10.1101/2020.03.13.990226

9. Ng OW, Chia A, Tan AT, Jadi RS, Leong HN, Bertoletti A, et al. Memory T cell responses targeting the SARS coronavirus persist up to 11 years post-infection. Vaccine. 2016;34: 2008–2014. doi:10.1016/j.vaccine.2016.02.063

10. Yamin D, Jones FK, DeVincenzo JP, Gertler S, Kobiler O, Townsend JP, et al. Vaccination strategies against respiratory syncytial virus. Proc Natl Acad Sci U S A. 2016. doi:10.1073/pnas.1522597113

11. Designed Research; S APGMM. Projecting hospital utilization during the COVID-19 outbreaks in the United States. 2020;117: 9122–9126. doi:10.1073/pnas.2004064117/-/DCSupplemental

12. Kissler SM, Tedijanto C, Goldstein E, Grad YH, Lipsitch M. Projecting the transmission dynamics of SARS-CoV-2 through the postpandemic period. Science (80- ). 2020;368: eabb5793. doi:10.1126/science.abb5793

13. Lauer SA, Grantz KH, Bi Q, Jones FK, Zheng Q, Meredith HR, et al. The Incubation Period of Coronavirus Disease 2019 (COVID-19) From Publicly Reported Confirmed Cases: Estimation and Application. Ann Intern Med. 2020. doi:10.7326/M20-0504

14. Linton NM, Kobayashi T, Yang Y, Hayashi K, Akhmetzhanov AR, Jung S, et al. Incubation Period and Other Epidemiological Characteristics of 2019 Novel Coronavirus Infections with Right Truncation: A Statistical Analysis of Publicly Available Case Data. J Clin Med. 2020;9: 538. doi:10.3390/jcm9020538

15. He X, Lau EHY, Wu P, Deng X, Wang J, Hao X, et al. Temporal dynamics in viral shedding and transmissibility of COVID-19. Nat Med. 2020;26: 672–675. doi:10.1038/s41591-020-0869-5

16. Gandhi M, Yokoe DS, Havlir D V. Asymptomatic Transmission, the Achilles’ Heel of Current Strategies to Control Covid-19. N Engl J Med. 2020. doi:10.1056/nejme2009758

17. Bendavid E, Mulaney B, Sood N, Shah S, Ling E, Bromley-Dulfano R, et al. COVID-19 Antibody Seroprevalence in Santa Clara County, California. medRxiv. 2020; 2020.04.14.20062463. doi:10.1101/2020.04.14.20062463

18. Gudbjartsson DF, Helgason A, Jonsson H, Magnusson OT, Melsted P, Norddahl GL, et al. Spread of SARS-CoV-2 in the Icelandic Population. N Engl J Med. 2020. doi:10.1056/nejmoa2006100

19. (No Title). Available: https://www.zva.gov.lv/sites/default/files/inline-files/05_07_covid-19-rapid-risk-assessment-coronavirus-disease-2019-ninth-update-23-april-2020-1.pdf

20. Czech study shows very low COVID-19 incidence in population. [cited 30 May 2020]. Available: https://medicalxpress.com/news/2020-05-czech-covid-incidence-population.html

21. Prem K, Liu Y, Russell TW, Kucharski AJ, Eggo RM, Davies N, et al. The effect of control strategies to reduce social mixing on outcomes of the COVID-19 epidemic in Wuhan, China: a modelling study. Lancet Public Heal. 2020;5: e261–e270. doi:10.1016/S2468-2667(20)30073-6

22. Anderson RM, Heesterbeek H, Klinkenberg D, Hollingsworth TD. How will country-based mitigation measures influence the course of the COVID-19 epidemic? The Lancet. Lancet Publishing Group; 2020. pp. 931–934. doi:10.1016/S0140-6736(20)30567-5

23. Zimmermann P, Curtis N. Coronavirus infections in children including COVID-19: An overview of the epidemiology, clinical features, diagnosis, treatment and prevention options in children. Pediatric Infectious Disease Journal. Lippincott Williams and Wilkins; 2020. pp. 355–368. doi:10.1097/INF.0000000000002660

24. Gaunt ER, Hardie A, Claas ECJ, Simmonds P, Templeton KE. Epidemiology and Clinical Presentations of the Four Human Coronaviruses 229E, HKU1, NL63, and OC43 Detected over 3 Years Using a Novel Multiplex Real-Time PCR Method. J Clin Microbiol. 2010;48: 2940–2947. doi:10.1128/JCM.00636-10

25. Wang J, Tang K, Feng K, Lv W. High Temperature and High Humidity Reduce the Transmission of COVID-19. SSRN Electron J. 2020. doi:10.2139/ssrn.3551767

26. Ficetola GF, Rubolini D. Climate affects global patterns of COVID-19 early outbreak dynamics. medRxiv. 2020; 2020.03.23.20040501. doi:10.1101/2020.03.23.20040501

27. Bock Axelsen J, Yaari R, Grenfell BT, Stone L. Multiannual forecasting of seasonal influenza dynamics reveals climatic and evolutionary drivers. [cited 24 May 2020]. doi:10.1073/pnas.1321656111

28. HOUSEHOLDS AND FAMILIES: DEMOGRAPHIC CHARACTERISTICS 2018 Based on Labour Force Surveys. [cited 8 Jun 2020]. Available: https://www.cbs.gov.il/en/publications/Pages/2020/HOUSEHOLDS-FAMILIES-LabourForce-2018.aspx

29. Mossong JL, Hens N, Jit M, Beutels P, Auranen K, Mikolajczyk R, et al. Social contacts and mixing patterns relevant to the spread of infectious diseases. PLoS Med. 2008;5. doi:10.1371/journal.pmed.0050074

30. Zhou F, Yu T, Du R, Fan G, Liu Y, Liu Z, et al. Clinical course and risk factors for mortality of adult inpatients with COVID-19 in Wuhan, China: a retrospective cohort study. Lancet. 2020;395: 1054–1062. doi:10.1016/S0140-6736(20)30566-3

31. Coronavirus (COVID-19) Data Dashboard - Novel Coronavirus (COVID-19) - County of Santa Clara. [cited 24 May 2020]. Available: https://www.sccgov.org/sites/covid19/Pages/dashboard.aspx

32. Italy Coronavirus: 232,664 Cases and 33,340 Deaths - Worldometer. [cited 30 May 2020]. Available: https://www.worldometers.info/coronavirus/country/italy/

33. Li W, Zhang B, Lu J, Liu S, Chang Z, Cao P, et al. The characteristics of household transmission of COVID-19. [cited 24 May 2020]. doi:10.1093/cid/ciaa450/5821281

34. Wei WE, Li Z, Chiew CJ, Yong SE, Toh MP, Lee VJ. Presymptomatic Transmission of SARS-CoV-2 — Singapore, January 23–March 16, 2020. MMWR Morb Mortal Wkly Rep. 2020;69: 411–415. doi:10.15585/mmwr.mm6914e1

35. Coronavirus disease (COVID-19): How is it transmitted? [cited 9 Feb 2021]. Available: https://www.who.int/emergencies/diseases/novel-coronavirus-2019/question-and-answers-hub/q-a-detail/coronavirus-disease-covid-19-how-is-it-transmitted

36. Dowell SF, Shang Ho M. Seasonality of infectious diseases and severe acute respiratory syndrome - What we don’t know can hurt us. Lancet Infectious Diseases. Lancet Publishing Group; 2004. pp. 704–708. doi:10.1016/S1473-3099(04)01177-6

37. Subjects - Live Births. [cited 8 Jun 2020]. Available: https://www.cbs.gov.il/en/subjects/Pages/Live-Births.aspx

38. Fertility Rate - the Haredi Institute for Public Affairs. [cited 10 Feb 2021]. Available: https://machon.org.il/en/series/fertility-rate/

39. COVID-19 Datasets- Government Data. [cited 30 May 2020]. Available: https://data.gov.il/dataset/covid-19

## 
